# Predictors of death including quality of positive pressure ventilation during newborn resuscitation and the relationship to outcome at seven days in a rural Tanzanian hospital

**DOI:** 10.1371/journal.pone.0202641

**Published:** 2018-08-17

**Authors:** Robert Moshiro, Jeffrey M. Perlman, Hussein Kidanto, Jan Terje Kvaløy, Paschal Mdoe, Hege L. Ersdal

**Affiliations:** 1 Department of Paediatrics and Child Health, Muhimbili National Hospital, Dar es Salaam, Tanzania; 2 Faculty of Health Sciences, University of Stavanger, Stavanger, Norway; 3 Department of Pediatrics, Weill Cornell Medicine, New York, NY, United States of America; 4 School of Medicine, Aga Khan University, Dar es Salaam, Tanzania; 5 Department of Mathematics and Physics, University of Stavanger, Stavanger, Norway; 6 Research Department, Stavanger University Hospital, Stavanger, Norway; 7 Department of Obstetrics and Gynecology, Haydom Lutheran Hospital, Manyara, Tanzania; 8 Department of Anesthesiology and Intensive Care, Stavanger University Hospital, Stavanger, Norway; Vanderbilt University Medical Center, UNITED STATES

## Abstract

**Background:**

Effective positive pressure ventilation (PPV) of non-breathing newborns is crucial in facilitating cardio-respiratory adaptation at birth. Identifying predictors of death in newborns receiving PPV is important in order to facilitate preventative strategies.

**Objective:**

The objective of this study was to determine the perinatal predictors of death including the quality of PPV administered among admitted newborns.

**Methods:**

An observational study of admitted newborns who received PPV after birth was conducted. Research assistants observed all deliveries and recorded perinatal events on data collection forms. Measured heart rate (HR) and ventilation parameters were then compared between newborns who died and survivors.

**Results:**

Newborns (*n* = 232) were studied between October 2014 and November 2016. Newborns who died (*n* = 53) compared to survivors (*n* = 179) had more fetal heart rate (FHRT) abnormalities (12/53 vs 19/179; *p* = 0.03); lower initial HR (<100 beats/minute) at start of PPV (44/48 vs 77/139; *p*<0.001); and a longer time for HR to increase >100 beats/minute from birth (180 vs 149 seconds; *p* = 0.07). Newborns who died compared to survivors took longer time (14 vs 4 seconds; *p* = 0.008) and more inflations (7 vs 3; *p* = 0.006) to achieve an expired volume (V_t_) of 6 ml/kg, respectively. Median delivered V_t_ during the first 60 seconds of PPV was less in newborns who died compared to survivors (5 vs 6 ml/kg; *p* = 0.12). Newborns who died proceeded to severe encephalopathy (15/31 vs 1/59; *p*<0.001) compared to survivors.

**Conclusion:**

Depressed newborns who proceeded to death compared to survivors, exhibited delayed HR response to PPV which may partly reflect FHRT abnormalities related to interruption of placental blood flow, and/or a timely delay in establishing adequate V_t_. Depressed newborns progressed to moderate/severe encephalopathy. Improving FHRT monitoring to identify fetuses at risk for expedited delivery, coupled with optimizing delivery room PPV might decrease mortality in this setting.

## Introduction

Approximately 23% of newborn deaths in Sub-Saharan Africa are related to intrapartum hypoxia/ischemia [1]. Transition from intra-uterine to extra-uterine life is a crucial phase for newborns. In Tanzania, approximately 15 percent of newborns will need some assistance to make this transition at birth; six percent require positive pressure ventilation (PPV) [2]. Therefore, provision of effective PPV is important to improve outcomes for those who fail to initiate spontaneous breathing at birth.

The aim of initial PPV is to establish functional residual capacity (FRC) [3]. The establishment of FRC is accompanied by an increase in pulmonary blood flow and an increase in heart rate (HR), indicating successful gaseous exchange [4]. Thus, an increase in HR is considered to be the most important clinical indicator of effective PPV, during newborn resuscitation [5,6]. The optimal volume during ventilation is unknown, however, an expired tidal volume (V_t_) between 3.3 and 10.5ml/kg is normally generated during spontaneous breathing at birth [7–9] and during face mask ventilation after birth [10,11]. We recently showed that a minimum V_t_ of 6 ml/kg is required to produce an increase in HR during PPV [12].

The commonest reason for a HR <100 beats/minute (BPM) upon delivery is secondary to an interruption of placental blood flow, often loosely termed “birth asphyxia”. If the interruption of blood flow is brief, most neonates are presumed to be in primary apnoea and will invariably respond to stimulation/suction with an increase in HR [12]. If the interruption in blood flow is more prolonged, the neonate is likely to be in secondary apnoea and will be less likely to respond to stimulation alone, invariably requiring PPV to correct the bradycardia. The International Liaison Committee of Resuscitation (ILCOR) recommends that PPV should be initiated on a newborn with a HR <100 BPM [6]. Few studies have documented changes in HR during PPV in relation to outcome as a measure of efficacy of ventilation. Most of the studies were performed in premature newborns and in the developed world [13,14].

When interruption of placental blood flow is prolonged or severe, the risk for hypo-perfusion to the brain is substantial. Such neonates will present with apnoea, and prolonged bradycardia, even with effective PPV [15]. The risk for subsequent hypoxic ischaemic encephalopathy (HIE) is markedly increased. Several postnatal factors are known to exacerbate on-going neuronal injury caused by birth asphyxia, including hypothermia/hyperthermia, seizures and hypoglycaemia [16,17]. Monitoring and targeted management strategies during the first hours after birth, is crucial for enhancing outcome of asphyxiated babies.

Investigating factors affecting outcome of newborns receiving PPV could provide important insight with regards to future resuscitation training, particularly as it relates to labour monitoring and assessment as well as the post-delivery care of newborns in low-resource settings. The objective of this study was to determine the perinatal predictors of death including the quality of PPV administered in the delivery room to newborns following admission to a newborn area.

## Methods

**Fig 1:**
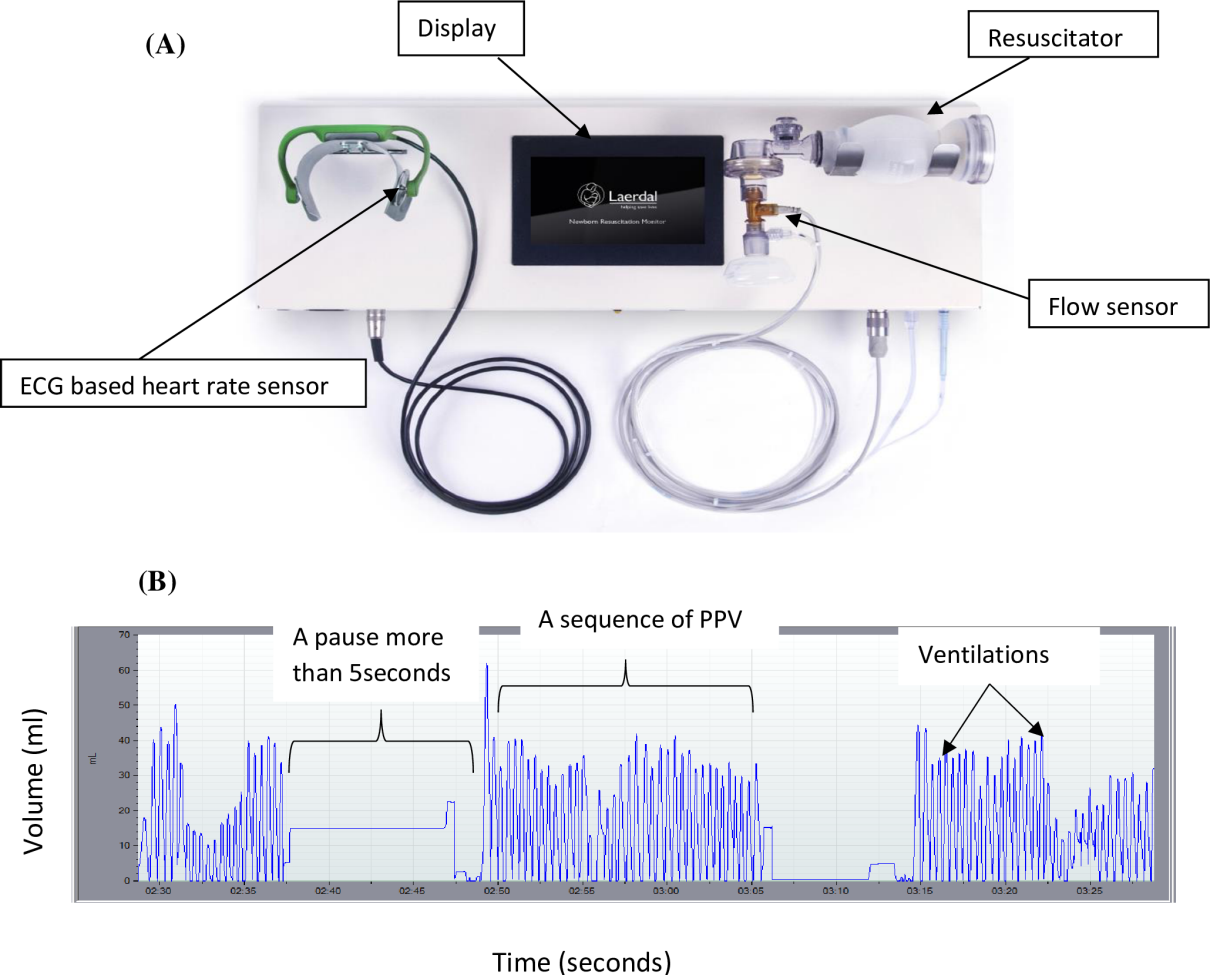
Newborn Resuscitation Monitor with a dry-electrode heart rate sensor, a resuscitator and signal output. (A) Monitor with ECG-based heart rate sensor and a resuscitator with flow sensor. (B) Volume signal curve extracted from the monitor.

This was an observational study on newborn resuscitation at Haydom hospital, a rural hospital with an estimated 4500 deliveries annually, providing comprehensive emergency obstetric care services 24 hours a day. Midwives conducted all deliveries including labour monitoring using the partograph as the assessment tool. Intermittent FHRT monitoring was performed using either a Pinard stethoscope or Doppler depending on the preference of the midwife. Normal FHRT was defined as between 120–160 BPM and FHRT persistently outside this normal range was considered abnormal. Newborn resuscitation was also conducted by midwives trained in Helping Babies Breathe, a resuscitation program tailored for the low resource setting [18]. Research assistants (*n* = 14) were trained to observe and document every delivery in the labour ward and theatre. All resuscitations were performed using Newborn Resuscitation Monitors (NRM, Laerdal Global Health, Stavanger, Norway; Fig 1), a research tool developed for the purpose of recording resuscitations.

The hospital’s newborn area accommodates 10–15 patients. Admission criteria include prematurity (gestational age <34 weeks), five-minute Apgar score <7, fever (>38°C) and signs of respiratory compromise, i.e., intercostal, subcostal retractions, or grunting. The newborn area offers antibiotics, oxygen therapy, phototherapy, and intravenous fluids. No mechanical ventilation or continuous positive airway pressure is available. From October 2014 to November 2016, admitted newborns who received PPV in the delivery room were consecutively enrolled, and followed-up until discharge or death within the first seven days. Information about admitted newborns in the newborn area was captured using a data collection form. Ventilated newborns who died in the delivery room were excluded from this study because they have been reported elsewhere [15].

### HR and expired tidal volume (V_t_) measurements

HR measurements were recorded and displayed on the screen in front of the providers by NRMs installed in all delivery rooms and the operating theatre. The monitor comprised a self-inflating bag without positive end expiratory pressure (PEEP), and an electrocardiogram (ECG) dry-electrode-based HR sensor [19]. The application of the ECG based sensor takes approximately 3 seconds to complete, and a HR is detected within approximately 5 seconds [19]. All newborn resuscitators were fitted with ventilation sensors between the bag and the mask, measuring flow, pressure and CO_2_. The flow sensors, using hot-wire anemometer technology, measured flow during expiration, and volume was calculated by flow signal integration. Mask leakage was calculated by the difference between inspiratory and expiratory volume. Time in seconds for HR to reach 100 BPM and V_t_, was downloaded from the NRM and extracted using Q-CPR Review 3.1.2.2 (Laerdal Medical, Stavanger, Norway). An episode of PPV was defined as time from the start of PPV to the end of PPV. Resuming PPV after a pause of >5 seconds was considered to be the start of a new ventilation sequence (Fig 1). Ventilation fraction was defined as time spent during actual ventilation, and ventilation frequency was defined as inflations per minute. We considered V_t_ of 6 ml/kg as the minimum volume required to increase HR during PPV [12]. We therefore defined quality of ventilation as a V_t_ of 6 ml/kg together with a rapid increase in HR above 100 BPM within 15 to 30 seconds after commencing PPV [4].

### Initial assessment during admission

Management of newborns followed the principles outlined in the WHO Essential Newborn Care guidelines [20]. Axillary temperature and blood glucose measurements were obtained using a digital thermometer and commercial glucometer, respectively, within 30 minutes of admission. Hypoglycemia, defined as blood glucose below 2.5 mmol/L [21], was treated with intravenous bolus 3 ml/kg 10% dextrose followed by feeding or maintenance with 10% dextrose. Severity of encephalopathy was classified using Thompson’s Hypoxic Ischaemic Encephalopathy (HIE) score [22]. Newborns scoring 1–10 were classified as mild HIE, 11–14 as moderate HIE, and 15–22 as severe HIE. Neonatal seizures were defined as paroxysmal, repetitive movements of the hands, face, feet, eyes or mouth. Reported seizures were those noted by the health care worker or the mother, both of whom may have not been present throughout. Seizures, if detected, were treated with intravenous Phenobarbital at a loading dose of 20 mg/kg, followed by maintenance dose of 5 mg/kg/day. Prophylactic antibiotics (ampicillin 50 mg/kg/day and gentamycin 4 mg/kg/day) were given to newborns with moderate and severely HIE for at least 48 hours because of the inability to exclude infections in this setting.

### Data analysis

Categorical data were summarized as numbers and percentages, and continuous data as means and standard deviations (SD) or medians and interquartile range (IQR) as appropriate. Chi-square tests were used to test for differences in categorical variables, and Mann-Whitney *U* or *t*-tests, as appropriate, were used to test for differences in continuous variables. Multivariable logistic regression analysis was then used to model how various variables influenced the risk of death. Variables with *p* <0.20 in the univariable analyses were included in the multivariable analysis using a stepwise backward elimination method, with the retention of predictors with *p* <0.05. The final retained variables were then entered into the model again and results are presented as Odds Ratio (OR) with 95% confidence intervals (CIs). Variables obtained on initial assessment during admission were entered into a separate model because of missing data. Statistical analyses were performed using SPSS (IBM SPSS Statistics for Windows, version 22.0; IBM Corp., Armonk, N.Y. USA).

### Ethical clearance

This study was approved by the National Institute of Medical Research in Tanzania and the Regional Committee for Medical and Health Research Ethics, Western Norway. All mothers gave verbal informed consent after being given information about the study by the attending midwife. The midwife then signed the consent on behalf of the mother. We were allowed to obtain verbal consent because some of the women came in labour and the high illiteracy rate in this area.

## Results

### General characteristics

**Fig 2:**
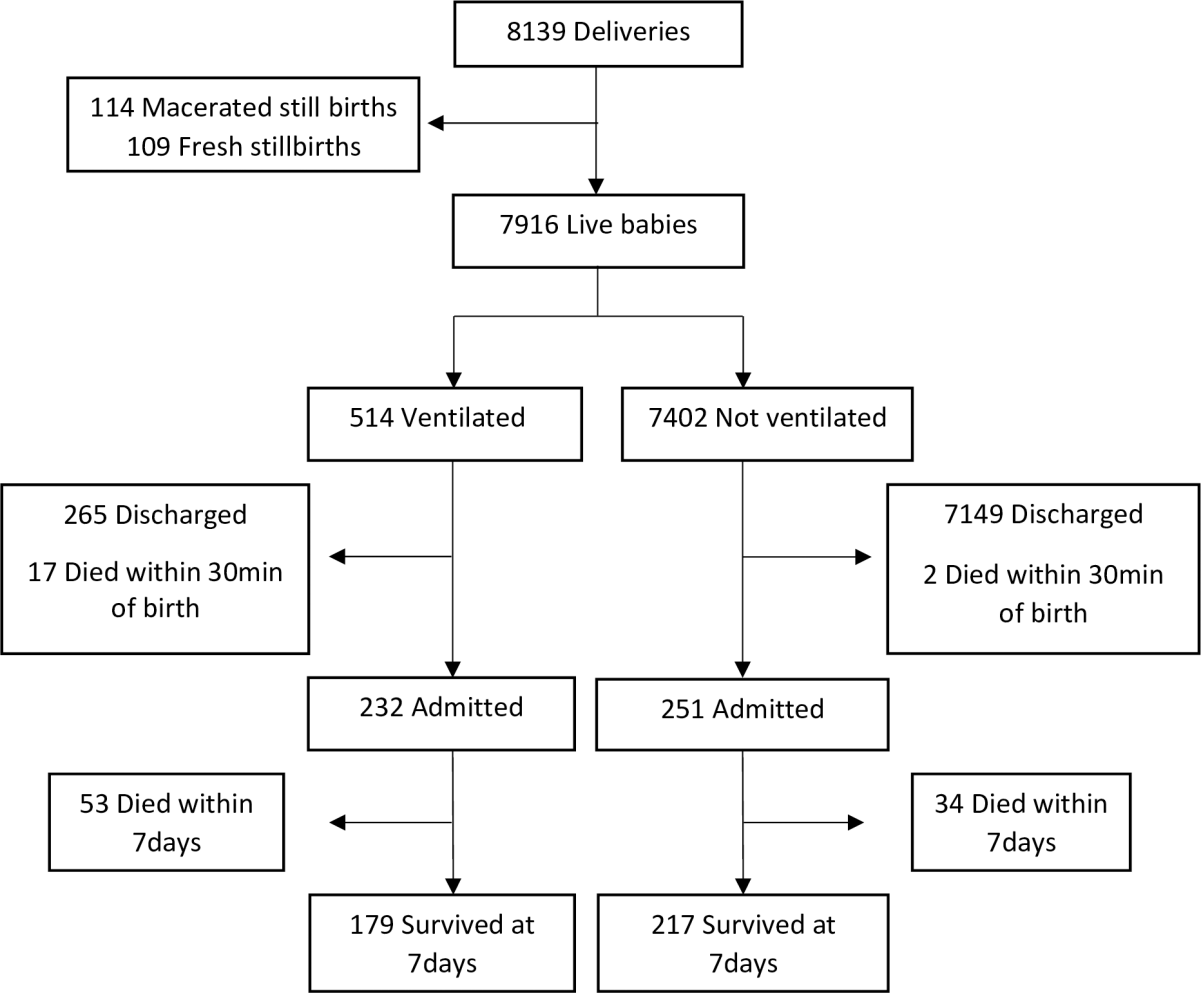
Flow diagram of patient recruitment.

During the study period, there were 8139 deliveries of mean birth weight 3287±529 grams and gestation age 39±2 weeks. A total of 1387 (18%) newborns received stimulation and/or suctioning, and 514 (6.3%) received PPV. Of these, 232 newborns were admitted and included in the study; 53 (23%) died within seven days (Fig 2). Of those who died, 15 (28%) died within 24 hours, and 75% died within the initial 72 hours of life. Among the newborns receiving PPV, 282 were not included; 17 died in the delivery room, and 265 recovered and stayed with the mother (were not admitted). The 17 newborns who died in the delivery room had mean birth weight 2931±551 grams and gestational age 37±3 weeks. The characteristics of this cohort have been reported previously [15]. Due to equipment malfunction (*n* = 15) and missing data (*n* = 21), ventilation parameters were only available for 196 admitted newborns (four of the 36 newborns with missing data died).

### Newborn characteristics and outcome of newborns who died versus survivors

Comparison of newborn characteristics between those who died and survivors through day 7 of life are shown in Table 1. Newborns who died as compared to survivors had more FHRT abnormalities during labour, 5-minute Apgar score <7 and a lower HR (<100 BPM) at the start of PPV. At the end of ventilation, 13/121 (11%) newborns had a HR <100 BPM, of whom 9 (70%) died. Newborns who died as compared to those who survived had significantly lower temperature, lower oxygen saturation, higher blood glucose, and progressed to severe encephalopathy (Table 1). Newborns who died were likely to have seizures, and to receive Phenobarbital and oxygen therapy. Of the newborns who died, 29/53 (55%) did so within first 24 hours of life. The most common presumptive cause of death was severe HIE. Mode of delivery (*p* = 0.35), gestational age (*p* = 0.20) and birthweight (*p* = 0.29) were comparable for both groups.

**Table 1:** Comparison of newborn characteristics among babies who survived versus dead within first 7 days.

	Survived *n* = 179	Dead *n* = 53	*P*-value
	*mean±SD* or *n* (%)	*mean*±*SD* or *n* (%)	
Gestational age (weeks)	38.0±2.5	37.0±3.0	0.20^b^
Birth weight (grams)	2910±600	2811±620	0.29^b^
FHRT during labour			
Normal	148 (83)	35 (66)	0.030^a^
Abnormal/not detected	19 (11)	12 (23)	
Not measured	12 (7)	6 (11)	
Apgar score at 5^th^ minute			
<7	34 (19)	24 (45)	<0.001^a^
≥7	145 (81)	29 (55)	
Initial HR recorded (BPM)	99 (64–148)	61 (53–80)	<0.001^c^
HR at the start of ventilation			
<100 BPM	77 (55)	44 (92)	<0.001^a^
≥100 BPM	62 (45)	4 (8)	
HR at the end of ventilation			
<100 BPM	4 (3)	9 (18)	<0.001^a^
≥100 BPM	134 (97)	39 (82)	
Initial assessment and treatment on admission			
Temperature (degree Celsius)^d^	35.2 ± 0.9	34.7 ± 1.0	0.004^b^
Oxygen saturation (%)^e^	81.0 ± 18.0	69.0 ± 22.0	0.004^b^
Oxygen supplement^f^	53 (62)	33 (92)	0.001^a^
Blood glucose (mmol/L)^g^	3.9 ± 2.1	4.6 ± 2.9	0.23^b^
HIE score on admission (*n* = 90)			
Normal	13 (22)	1 (3)	<0.001^a^
Mild	38 (64)	9 (29)	
Moderate	7 (12)	6 (19)	
Severe	1 (2)	15 (48)	
Seizures (*n* = 124)			
Yes	9 (10)	15 (40)	<0.001^a^
No	78 (90)	22 (50)	
Phenobarbital	26 (30)	20 (54)	0.012^a^

FHR, fetal heart rate; SVD, spontaneous vaginal delivery; CS, caesarean section; HR, heart rate; BPM, beats/minute.

^a^chi-square.

^b^t-test.

^c^Mann-Whitney *U* test.

^d^missing value *n* = 127.

^e^missing value; *n* = 103.

^f^missing value *n* = 109.

^g^missing value; n = 1.

### HR responses and ventilation parameters in relation to outcome

Factors related to newborn responses to PPV in relation to outcome are summarized in Table 2. Newborns who died compared to those who survived took a longer time to reach HR >100 BPM (*p* = 0.007). Newborns who died compared to those who survived received less volume (ml/kg) during the first 60 seconds of PPV, i.e., 5 versus 6 (*p* = 0.13), and more volume during the whole episode of PPV, i.e., 9 versus 7 (*p* = 0.41) (Table 2). It took a longer time to achieve a V_t_ ≥6 ml/kg for those newborns who died compared to those who survived, i.e., a median value of 14 versus 4 seconds (*p* = 0.008), respectively. The number of ventilations to achieve a TV >6 ml/kg was greater in the infants who died compared to those who survived i.e., median of 7 vs 3 (*p* = 0.006). Newborns who died were ventilated for a much longer time compared to survivors, i.e., 402 versus 230 seconds (*p* = 0.001), respectively (Table 2). There was less mask leakage in newborns who died compared to those who survived (*p* = 0.027).

**Table 2:** Responses of newborns to positive pressure ventilation in relation to outcome survived vs dead within first 7 days.

Variables	Survived n = 179 Median (IQR) or *n*(%)	Dead n = 53 Median IQR or *n*(%)	*P*-value
Stimulation (yes)	179 (100%)	53 (100%)	
Suction (yes)	176 (98%)	44 (83%)	0.92^b^
Time from birth to cord clamp (s)	25 (11–57)	18 (10–38)	0.19^a^
Time from birth to application of HR sensor (s)	108 (76–158)	116 (73–140)	0.52^a^
Time from birth to detection of initial HR (s)	113 (85–158)	116 (83–152)	0.92^a^
Time from birth to start PPV (s)	115 (85–17)	117 (85–147)	0.35^a^
Time from birth to HR increase >100 BPM (s)^c^	149 (105–208)	180 (119–235)	0.07^a^
V_t_ first 60s of PPV (ml/kg)	6 (4–12)	5 (3–10)	0.12^a^
V_t_ whole episode of PPV (ml/kg)	8 (5–12)	9 (5–14)	0.36^a^
Vol/min first 60s of PPV (ml/min)	896 (429–1528)	781 (375–1414)	0.14^a^
Vol/min whole episode of PPV (ml/min)	1140 (643–1876)	1389 (731–1900)	0.41^a^
Time from start PPV to V_t_ >6 ml/kg (s)	4 (1–18)	14 (2–31)	**0.008**^a^
Number of ventilations before >6 ml/kg	3 (1–10)	7 (2–20)	**0.006**^a^
Time used ventilating during first 60s (s)	40 (28–55)	44 (31–54)	0.57^a^
PPV frequency during first 60s (inflations/minute)	45 (37–57)	50 (40–59)	0.19^a^
PPV frequency during whole episode (inflations/minute)	44 (36–55)	52 (39–60)	0.25^a^
Mask leak during ventilation (%)	40 (26–54)	33 (21–55)	**0.027**^a^
Time of whole episode of PPV (s)	230 (104–466)	402 (203–785)	**<0.001**^a^

PPV, positive pressure ventilation; Vol/min, volume per minute; s, seconds; HR, heart rate; BPM, beats/minute; V_t_, expired tidal volume; IQR, inter-quartile range.

^a^Mann-Whitney *U* tests.

^b^chi-square.

All ventilation parameters *n* = 196 except ^c^median time from birth to HR to increase >100 BPM *n* = 127.

Results of the univariable and multivariable analyses are shown in Tables 3 and 4. Abnormal FHRT (*p* = 0.018), 5-minute Apgar score <7 (*p*≤0.001), HR at the start of PPV (*p*≤0.001), HR at the end of PPV (*p*≤0.001), mask leakage (*p* = 0.026) and median time of PPV (*p*≤0.001), were all independently associated with increased odds of death (Table 3). When adjusted for all variables in the final model, abnormal FHRT (*p* = 0.019), HR at the end of PPV (*p* = 0.002) and median duration of PPV (*p* = 0.001) were all associated with increased odds of death. Postnatal predictors of death are summarized in Table 4. Admission temperature (*p* = 0.003), seizures (*p*<0.001), oxygen saturation (*p* = 0.003) and moderate/severe HIE (*p*<0.001) were independently associated with death within first seven days. When adjusted for the rest of the variables in the model, moderate/severe HIE (*p*<0.001) was significantly associated with risk of death.

**Table 3:** Logistic regression model for independent predictors of death within first 7 days among admitted newborns.

Variables	Univariable analysis OR (95% CI)	*P* value	Multivariable analysis with all variables into the model AOR (95% CI)	*P* value	Multivariable analysis after backward LR elimination AOR (95% CI)	*P* value
FHRT during labour						
Normal	1		1		1	
Abnormal/not detected	2.67 (1.19–6.01)	0.018	3.94 (1.25–12.40)	0.019	3.15 (1.21–8.19)	0.018
Not measured	2.11 (0.74–6.02)	0.16	1.29 (0.23–7.11)	0.77	3.09 (0.93–10.23)	0.06
Apgar score						
≥7	1		1			
<7	3.53 (1.83–6.81)	<0.001	2.19 (0.84–5.01)	0.11		
HR at start of PPV						
≥100	1		1			
<100	8.86 (3.02–25.99)	<0.001	1.85 (0.18–19.63)	0.61		
HR at end of PPV						
≥100 BPM	1		1		1	
<100 BPM	7.54 (2.20–25.78)	0.001	5.94 (1.08–32.53)	0.040	7.63 (2.05–28.41)	0.002
Time from birth to HR increase >100BPM (s)	1.003 (0.99–1.00)	0.13	1.002 (0.99–1.01)	0.44		
Time to achieve 6 ml/kg (s)	1.00 (0.99–1.01)	0.18	1.00 (0.98–1.02)	0.97		
Mask leak (%)	0.98 (0.97–0.99)	0.026	0.98 (0.96–1.01)	0.37		
Median time of PPV (s)	1.002 (1.001–1.003)	<0.001	1.001 (1.00–1.002)	0.15	1.002 (1.001–1.003)	0.001
No ventilation before 6 ml/kg (s)	1.01 (0.99–1.02)	0.06	1.01 (0.98–1.03)	0.45		

PPV, positive pressure ventilation; FHRT, fetal heart rate; HR, heart rate; s, seconds; BPM, beats/minute; AOR, adjusted odds ratio; CI, confidence interval; LR, logistic regression.

**Table 4:** Logistic regression model for independent post-natal predictors of death within first 7 days among admitted newborns.

Variables	Univariable analysis OR (95% CI)	*P* value	Multivariable analysis with all variables into the model AOR (95% CI)	*P* value	Multivariable analysis after backward LR elimination AOR (95% CI)	*P* value
Admission temperature	0.49 (0.30–0.78)	0.003	0.64 (0.28–1.47)	0.29		
Oxygen saturation	0.97 (0.95–0.99)	0.003	0.99 (0.95–1.04)	0.68		
Blood glucose	1.13 (0.93–1.38)	0.20	1.26 (0.88–1.8)	0.20		
Seizures	5.91 (2.28–15.3)	<0.001	3.00 (0.48–18.8)	0.24		
HIE						
Normal/Mild	1		1		1	
Moderate/severe	13.0 (4.74–39.40)	<0.001	10.5 (2.12–52.8)	0.004	13.0 (4.74–39.40)	<0.001

HIE, hypoxic ischaemic encephalopathy; AOR, adjusted odds ratio; CI, confidence interval; LR, logistic regression.

## Discussion

The data in this report demonstrated that newborns who died within the first week of life as opposed to survivors exhibited more FHRT abnormalities in utero, lower initial HR as well as a lower HR following PPV, and received a longer duration of PPV. In addition, newborns who died were more likely to be depressed at birth, have a lower body temperature on admission to the neonatal area, and progress to moderate/severe encephalopathy and seizures.

The findings that newborns who died were more likely to exhibit an abnormal FHRT pattern during labour, present with lower HR at the initiation of ventilation, remain with a HR <100 BPM at the end of ventilation, and require PPV for a longer period strongly supports the mechanism of intrapartum asphyxia (interruption of placental blood flow) as a major cause of these cardio-respiratory findings. Three reasons may account for the slower HR response to ventilation. First, the newborns may have been in secondary apnoea with profound acidemia and a stunned myocardium. Second, ventilation was performed with a self-inflating bag without PEEP, which may have limited the ability to establish FRC; the latter has been shown to be intimately related to an increase in HR [12]. Third, quality of PPV may have been inadequate as there was a significant delay in achieving a targeted V_t_ of 6 ml/kg in newborns who died, likely contributed to by low lung compliance of newborns in secondary apnoea.

Several factors may have contributed to the quality of delivered inflations. First, Skare et al. noted that 50 percent of ventilated newborns had a ventilation fraction of 60% during the first 30 seconds of ventilation, indicating interruption rather than continuous PPV [23]. Newborns in our study had a similarly low ventilation fraction of 66% during first 60 seconds. This is inconsistent with current HBB guidelines, which recommend uninterrupted ventilation until spontaneous breathing begins [18]. Interruptions of PPV may result in derecruitment and may have contributed to further delays in achieving FRC. Furthermore, the delay in initiating PPV was well beyond the recommended time of within 60s after birth, termed the “Golden Minute” [18]. We have reported previously that for every 30-second delay in initiating ventilation, there is a 16% increased risk of death [2]. Although the delay in initiating ventilation was comparable in both groups, it may still have influenced the time to establish FRC. Second, the inadequate volume that was delivered during the first 60s, coupled with the increased number of ventilations needed to achieve a desired goal of 6 ml/kg, likely led to prolonged time to establish FRC in the newborns who died [12]. Whether this reflects the use of a self-inflating bag without PEEP, or relates to ventilation technique remains unclear. Third, mask leak was substantial (range of 40 to 50%) in both groups although less in those who died versus survivors. However, despite the interrupted PPV and significant mask leakage, newborns were still able to receive adequate V_t_ during the whole episode of PPV. This finding concurs with other human and mannequin studies indicating that despite mask leak, delivered tidal volume is unaffected [10,24,25]. Interestingly, as noted above, newborns who died had less mask leakage as compared to survivors. We speculate that the prolonged PPV among the babies who died may have resulted in improvement of ventilation technique and less mask leak over time. Despite all these factors that may have contributed to a poorer quality, PPV was not a significant predictor of death in the final analytical model.

Moderate/severe HIE was the most important postnatal predictor of death. The findings of an abnormal FHRT, immediate bradycardia upon delivery, and the more sustained bradycardia with PPV, strongly supports the interruption of placental blood flow as the proximate cause of the brain injury. To our knowledge, this is the first time that intrapartum events have been closely linked to early severe encephalopathy in a resource-limited setting. An additional factor that may have contributed to the severe brain injury was the hypothermia noted at the time of admission to the newborn area. This may have affected cardiac function and brain perfusion. Hypothermia also decreases cerebral metabolic rate, which, in turn, reduces cerebral utilization of glucose.

Hypothermia has been shown to increase mortality in newborns, and in particular those of low birth weight, possibly through its well-described negative effects on respiratory function, i.e., inhibiting the action of surfactant, and an increased risk of sepsis [26]. Furthermore, hypothermia in the context of associated birth asphyxia has been reported to increase the risk of death in a dose-dependent manner [27,28]. This is in contrast to the neuro-protective role of controlled therapeutic hypothermia in the management of newborns with encephalopathy in resource-replete countries [29]. Nevertheless, interpretation of the findings regarding hypothermia in this study should be done cautiously due to significant missing data.

This study was carried out in a typical rural hospital with many neonatal deaths due to birth asphyxia. The ability to measure and rapidly detect the HR during PPV and link it to events that occurred before birth is a major strength of this study. In addition, we were able to study a group of high-risk asphyxiated newborns, and to document the HR responses to PPV.

The limitations of this study include the possibility that gaseous exchange did not take place in some neonates, despite the finding of adequate expired volume. In addition, all ventilations were performed without PEEP, which could in part explain the delayed observed HR response. Furthermore, we had significant missing data on some of the variables. Finally, it is also important to acknowledge that the newborns who died in this low-resource setting may have survived without neurologic injury in a setting where advanced newborn care is available.

## Conclusion

This study showed that newborns depressed at birth with bradycardia, and with subsequent progression to death, had significantly delayed HR response to PPV as compared to survivors. Newborns who exhibited delayed HR response were also observed to have abnormal FHRT patterns, low Apgar scores and bradycardia at birth, likely related to intrapartum asphyxia. These infants went on to present with moderate/severe encephalopathy and seizures, the former being a significant predictor of death. Although the quality of PPV was not a significant predictor of death in the final analysis, it could have influenced outcome through delayed establishment of FRC. Finally, measures to improve FHRT monitoring during labour, and identification of those fetuses at high risk of severe brain injury, might help to decrease mortality due to perinatal asphyxia in the resource-limited setting.

## Supporting information

S1 Data File (SAV)
