# Correction: Predictors of death including quality of positive pressure ventilation during newborn resuscitation and the relationship to outcome at seven days in a rural Tanzanian hospital

**DOI:** 10.1371/journal.pone.0204084

**Published:** 2018-09-12

**Authors:** Robert Moshiro, Jeffrey M. Perlman, Hussein Kidanto, Jan Terje Kvaløy, Paschal Mdoe, Hege L. Ersdal

There are errors in the Funding and Competing interests sections. The following information is missing from the Funding section: The study was funded by the Laerdal Foundation.

The correct Competing interests information is as follows: The authors have declared that no competing interests exist.
